# Investigating the causes and pattern of peritoneal involvement in CT scan and comparison with ultrasound findings in peritoneal conditions: A two‐center, cross‐sectional study

**DOI:** 10.1002/hsr2.70026

**Published:** 2024-08-28

**Authors:** Farzaneh Khoroushi, Lida Jarahi, Ehsan Hassannejad, Nafise Kazemirad

**Affiliations:** ^1^ Department of Radiology Faculty of Medicine, Mashhad University of Medical Sciences Mashhad Iran; ^2^ Community Medicine Department Faculty of Medicine, Mashhad University of Medical Sciences Mashhad Iran; ^3^ Department of Radiology School of Medicine, Birjand University of Medical Sciences Birjand Iran; ^4^ Department of Radiology School of Medicine, Mashhad University of Medical Sciences Mashhad Iran

**Keywords:** CT scan, omentum involvement, peritoneal involvement, ultrasound

## Abstract

**Background and Aims:**

Ultrasound and computed tomography (CT) scans can be used as methods to help make a more accurate diagnosis in diseases involving the omentum and peritoneum. The present study was conducted to determine etiology and CT scan pattern of peritoneal involvement and compare it with ultrasound findings.

**Methods:**

Patients referred to Ghaem and Imam Reza hospitals who had undergone CT scans and had involvement of peritoneum in abdominal CT scans were included in the study. The findings identified in the CT scan were recorded. According to the underlying cause determined by laparotomy or laparoscopy sample, each disease's most common pattern of involvement and types of patterns were examined. An ultrasound was conducted for every patient and the obtained information was analyzed.

**Results:**

A total of 101 patients were included in the study, of which 61 patients (59.8%) were female and the rest were male. The most common involvement patterns in CT scans included nodular (37.6%), mixed (21.8%), and omental cake (17.8%), respectively. In 80 patients (79.2%), CT scan findings were consistent with ultrasound, but in 21 patients (20.8%), CT scan findings were not visible in ultrasound. The most common diagnoses of the patients were colorectal adenocarcinoma and gastric adenocarcinoma (20 cases, 19.8% each), followed by ovarian and uterine adenocarcinoma (19 cases, 18.8%).

**Conclusion:**

The most common patterns of involvement of peritoneum in CT scans include nodular pattern, mix, and omental cake. The most common diseases that lead to the involvement of peritoneum are gastrointestinal cancers, uterine and ovarian cancers, and peritonitis.

## 1 INTRODUCTION

Peritoneal carcinomatosis refers to metastatic implants in the visceral peritoneal lining, which can arise from several malignant causes. The prevailing causes include neoplasms originating from the colon, rectum, ovaries, stomach, and pancreas. In some instances, however, the tumor's origin may be unknown.^1^ Certain nonmalignant causes might exhibit similarities to peritoneal carcinomatosis, as shown in widespread peritoneal endometriosis, leiomyomatosis, and tuberculosis (TB).^2^ However, it is imperative for radiologists to identify this entity because of its substantial consequences in the staging, treatment, and prognosis of malignant patients. Furthermore, the radiologist must endeavor to ascertain the initial tumor in such cases, as this aids in the characterization of the genesis of peritoneal involvement.

The diagnostic accuracy of abdominal radiography in identifying peritoneal carcinomatosis is limited, as indicated by previous research.^3^ Exploratory laparoscopy remains the widely accepted benchmark for assessing and measuring peritoneal diseases. The method mentioned above is intrusive, presenting inherent difficulties and potential incompleteness caused by adhesions, which could be accompanied by complications.^4^ On the other hand, ultrasound imaging can identify hypoechoic masses in omental deposits and iso‐ to hyperechoic masses in the presence of ascites.^5^ Nevertheless, the use of ultrasonography may be restricted when dealing with tiny quantities of ascites and is subject to the skill and expertize of the operator. There is little doubt that computed tomography (CT) is the preferred imaging technique for assessing peritoneal carcinomatosis.^6^ The sensitivity of CT is contingent upon the size of the implant; it can be as high as 90% for lesions larger than 5 cm and as low as 25% for lesions with a size smaller than 0.5 cm.^7^

The present study assessed the causes and patterns of peritoneal involvement in CT scan and compared these findings with those of ultrasound.

## 2 METHODS AND MATERIALS

The present study was a cross‐sectional study on patients referred to Ghaem and Imam Reza Hospitals in Mashhad between 2018 and 2022 and had undergone an abdominal CT scan (either in the mentioned centers or outside of it) and had involvement of the peritoneum and omentum. All patients aged 18 years and above and obesity was also considered as body mass index ≥ 30. Exclusion criteria included lack of access to the complete CT scan file and lack of access to the patient for ultrasound. Then, a blinded expert radiologist, unaware of the diagnoses, operated ultrasonography of the included patients. First, the pattern of involvement (smooth, irregular, and nodular) was determined, and the associated findings identified in the CT scan, such as lymphadenopathy, splenomegaly, ascites, densitometry, and so forth, were recorded. The checklist of involvement patterns in CT and ultrasound included these items:

Increased omental thickness
Omental cake
Increased smooth thickening of the peritoneum
Increased irregular thickening of the peritoneum
Nodularity of the peritoneum

The cause of peritoneal involvement was determined according to the laparotomy or laparoscopy sample. Then, according to the underlying cause, each disease's most common pattern of involvement and types of patterns were examined. Also, every patient was called for an ultrasound conducted by a single blinded expert radiologist. In ultrasound, findings of involvement of peritoneum and omentum, as well as accompanying findings such as ascites and the presence of debris in it, lymphadenopathy, splenomegaly, etc., were recorded and compared with the findings of the patient's CT scan. Then, the patients were followed up by checking the postsurgery and post‐biopsy (pathology) findings, which is the standard method of diagnosing the disease, and the common findings of any disease involving the peritoneum were examined. It should be noted that the patients included in the study were randomly subjected to ultrasound along with other hospital patients, and the radiologist did not know which patients were under study. The time interval between CT scan imaging, laparoscopy and ultrasonography was at most 3 month.

**Figure 1:**
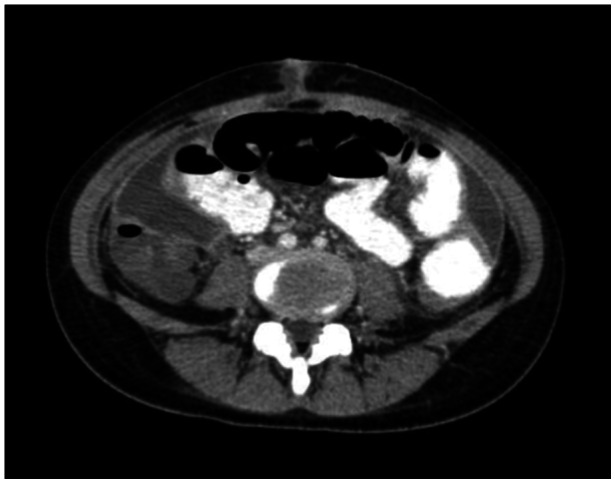
Increased smooth thickening of the peritoneum in CT scan. CT, computed tomography.

**Figure 2:**
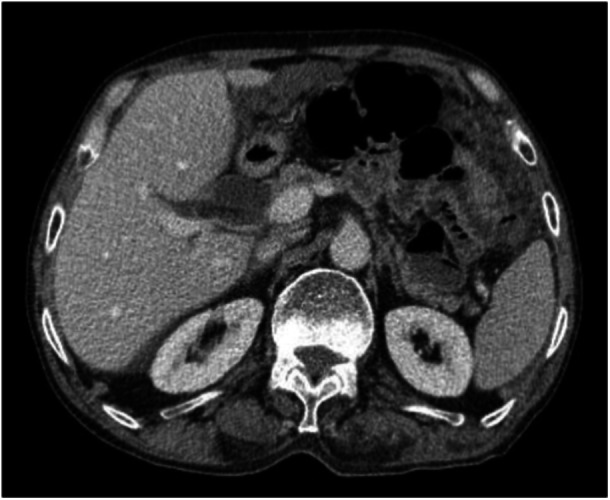
Nodularity of the peritoneum in CT scan. CT, computed tomography.

Figures 1 and 2 illustrate increased smooth thickening and nodularity of the peritoneum, respectively.

### 2.1 Ultrasound examination

The ultrasound examinations in the present study were conducted with the LOGIQ 9 ultrasound system manufactured by GE Healthcare, based in Milwaukee, WI, USA. Transvaginal ultrasound imaging was performed using vaginal probes with frequencies ranging from 9 to 3 MHz or 8 to 4 MHz, while transabdominal ultrasound imaging utilized probes with frequencies ranging from 5 to 2 MHz. The scans were directed toward the omentum, hepatic and splenic surfaces, diaphragm, lateral peritoneum, and bowel surface. A thorough examination of the mesenteric and intestinal surfaces was conducted upon observing ascites. The study involved documenting the size, echo, and color Doppler flow imaging of peritoneal implants in the pelvic and abdominal regions. Additionally, the boundaries and interactions between these implants and lesions in the surrounding tissues were documented. Simultaneous saving of imaging data was performed. The presence of a peritoneal carcinomatosis within the abdominal cavity was meticulously documented, taking into account the specific regions affected. These encompassed the surfaces of the colon, liver, spleen, diaphragm, lateral peritoneum, omentum, mesentery, and pelvis. The procedure employed for the examination of patients for polycystic disease using ultrasonography was as follows:

In the context of pelvic implants, transvaginal ultrasonography was employed to assess several anatomical structures, including the pouch of Douglas, pelvic parietal peritoneum, and surfaces of visceral peritoneum. This diagnostic technique facilitated the identification of lesions present on the surfaces of the colon, bladder, uterus, and pelvic parietal peritoneum.
The omentum was seen via abdominal ultrasonography, which involved imaging the upper and lower abdomen in either the longitudinal or transverse orientation. The lesions were often situated inside the interstitial space between the front abdominal wall and the gastrointestinal tract. The patient was positioned in a supine posture to examine the hepatic surface. Initially, a transverse scan of the left liver was conducted at the subxiphoid level.
Subsequently, a diagnostic procedure, including an oblique scan, was conducted along the right costal edge of the liver, proceeding from left to right. This was followed by an intercostal oblique and coronal scan of the liver surface while the patient was positioned in the left lateral position.
The diaphragm surface was examined by scanning the right costal border at an oblique angle, as well as by obtaining intercostal oblique and coronal sections in the left lateral position.
Lateral repositioning of the patient was performed to expose the splenic surface. The spleen can be examined using intercostal or left coronal scanning techniques, wherein the probe is maneuvered in a backward and forward motion.
The patient was positioned supine, with attention given to the mesenteric and intestinal surfaces. The presence of hyper‐echoic bowel can be noticed in all four quadrants. The operator searched for implants on the surfaces of the mesentery and bowel during the scanning process.
The patient was positioned in either the right or left lateral decubitus. The abdominal region on either the left or right side, situated close to the corresponding lateral aspect of the abdominal wall, was subjected to a top‐to‐bottom scanning procedure.

### 2.2 CT examination

The study utilized GE 64‐slice helical scanners (GE Medical Systems) to conduct CT scans. The images underwent reconstruction with a spacing of 7 mm millimeters between each interval. The intravenous contrast medium Ultravist (Bayer) was administered by delivering a bolus injection into the forearm vessel at a rate of 3 mL/s using a high‐pressure syringe. After administering the contrast agent, pictures were acquired at several time intervals, namely at 30 s for the arterial phase, 60 s for the portal phase, and 200 s for the delayed phase. The scanning range encompassed the region extending from the superior aspect of the diaphragm to the pubic symphysis. Every physician documented a diagnosis for each CT scan.

The preoperative descriptions of ovarian cancer by abdominal pelvic CT encompassed various aspects such as the size, morphology, and whether the ovarian masses were unilateral or bilateral. Any features indicating malignancy, such as invasions or implants in the uterus, bladder, bowel, or pelvic side wall, were also noted. The presence and volume of ascites in the pelvis or upper abdomen, as well as metastases in the omentum, small bowel mesentery, and surface of the bowel, were also documented. Furthermore, the presence of metastases on the surface or parenchyma of the liver and spleen, as well as implants in the para‐colic gutters and other peritoneal/serosal sites outside the pelvis, were recorded. Lastly, the locations of lymph nodes with short‐axis diameters greater than 1 cm or suspicious clusters of smaller lymph nodes were identified. This study did not encompass the examination of metastases in lymph nodes, hepatic parenchyma, and splenic parenchyma.

### 2.3 Statistics

The data was entered into SPSS 22 software. Descriptive analysis was expressed as frequency/percentage/central and dispersion indices. For analytical analysis, an independent *t*‐test was used for independent quantitative variables, and a paired *t*‐test was used for dependent quantitative variables. The chi‐square test was used to compare qualitative variables. A *p* Value of less than 0.05 was deemed to indicate statistical significance.

### 2.4 Ethics

All stages of the study followed the ethical principles of Helsinki and were approved by the Ethics Committee of Mashhad University of Medical Sciences with code IR.MUMS. MEDICAL.REC.1400.704. Informed consent was obtained from all patients to enter the study and Patient data were entered anonymously to remain confidential.

## 3 RESULTS

The study had a sample size of 101 patients, with 61 patients (59.8%) identified as female and the others as male. Furthermore, out of the total sample size, 33 individuals, accounting for 32.7% of the population, were classified as obese.

The results of the CT scan analysis conducted on the study population indicated that 70.3% of individuals exhibited evident ascites, while 14.9% had dense ascites, and an additional 14.9% did not exhibit any signs of ascites. Lymphadenopathy was identified in 40.6% of the examined instances, while metastasis was found in 32.7%. Additionally, organomegaly was discovered in 1% of the cases. Furthermore, calcification was seen in 2% of the instances, while 16.8% of the patients had a primary tumor. The distribution of involvement exhibited mass, nodule, or cyst in 44.5% of instances, mixed patterns in 21.7%, omental cake in 17.8%, smooth involvement in 12.8%, and haziness in 2.9%. The distribution of involvement exhibited variability, with 41.6% demonstrating diffuse involvement, 18.3% displaying mixed involvement, 12.5% localized in the left upper quadrant (LUQ), 11.6% localized in the right upper quadrant (RUQ), and 10% localized in the pelvis (Table 1).

**Table 1:** The findings of CT scan in the study population.

Characteristic		Frequency	%
Ascites	Clear	71	70.3
	Dense	15	14.9
	No ascites	15	14.9
Lymphadenopathy		41	40.6
Metastasis		33	32.7
Organomegaly		1	1
Calcification		2	2
Primary tumor		17	16.8
Pattern of involvement	Mass, nodule, or cyst	45	44.5
	Mixed	22	21.7
	Omental cake	18	17.8
	Smooth	13	12.8
	Haziness	3	2.9
Location of involvement	Diffuse	50	41.6
	Mixed	22	18.3
	LUQ	15	12.5
	RUQ	14	11.6
	Pelvis	12	10
		7	5.8

*Note*: The data are presented in frequency and %.

Abbreviations: CT, computed tomography; LUQ, left upper quadrant; RUQ, right upper quadrant.

The ultimate diagnosis of the research subjects revealed a wide array of illnesses. The most often seen types of cancer were colorectal adenocarcinoma, gastric adenocarcinoma, and adenocarcinoma of the ovaries/uterus accounting for 19.8%, 19.8%, and 18.8% of the total cases, respectively. GIST and peritonitis incidence rates were 2% and 10.9% in the studied patients, respectively. Additional diagnoses encompassed a range of malignant neoplasms, including esophageal adenocarcinoma, pancreatic adenocarcinoma, testicular rhabdomyosarcoma, breast cancer, sarcoma affecting the uterus, sarcomatoid mesothelioma, small cell lung cancer, adenocarcinoma affecting the appendix and ileum, cholangiocarcinoma, gestational trophoblastic neoplasia, hepatocellular carcinoma, lymphoma, adenocarcinoma and transitional cell carcinoma affecting the urinary tract, TB, squamous cell carcinoma teratoma, and ovarian fibrothecoma (Table 2).

**Table 2:** The final diagnoses of the study participants.

Diagnosis	Frequency	%
Colorectal adenocarcinoma	20	19.8
Gastric adenocarcinoma	20	19.8
Adenocarcinoma of the ovaries/uterus	19	18.8
Sarcoma of the uterus	5	5
Breast cancer	1	1
Ovarian fibrothecoma	1	1
GTN	1	1
GIST	2	2
Peritonitis	11	10.9
Esophageal adenocarcinoma	1	1
Pancreatic adenocarcinoma	3	3
Testicular rhabdomyosarcoma	1	1
Sarcomatoid mesothelioma	1	1
Small cell lung cancer	1	1
Adenocarcinoma of the appendix/ilium	2	2
Cholangiocarcinoma	3	3
HCC	1	1
Lymphoma	2	2
Adenocarcinoma/TCC of the urinary tract	3	3
TB	2	2
SCC teratoma	1	1

*Note*: The data are presented in frequency and %.

Abbreviations: GIST, gastrointestinal stromal tumor; GTN, gestational trophoblastic neoplasia; HCC, hepatocellular carcinoma; SCC, squamous cell carcinoma; TCC, transitional cell carcinoma; TB, tuberculosis.

The present study also analyzed the correlation between the gender of patients and the results of CT scans. The analysis reveals a notable correlation between the patient's gender and the location of involvement. Specifically, male patients saw 30% of the conflict cases in the LUQ. The results showed a significant disparity between the male and female genders with this matter (*p* = 0.001).

The analysis of the correlation between obesity in patients, as determined by CT scan data, and the ultimate diagnosis of these individuals showed no statistically significant correlation between the patient's obesity and the parameters under investigation (*p* > 0.05).

The correlation between CT scans and ultrasounds demonstrated concordant results in relation to gender and obesity. Moreover, notable disparities were seen in some instances when comparing CT scan findings. It is worth noting that a statistically significant correlation was seen between gender and CT scan results (*p* = 0.03), whereas 55% of females and 45% of men exhibited concordant instances. Furthermore, the CT pattern had a strong and statistically significant association with matching (*p* < 0.001). Some patterns, such as omental cake, notably demonstrated elevated matching rates. The observed variations in the site of participation did not reach statistical significance (Table 3).

**Table 3:** The association between CT scan and ultrasound matched findings with gender, obesity, and CT scan findings.

Characteristic		Nonmatched cases	Matched cases	*p* Value
Gender	Female	17 (81)	44 (55)	0.03
	Male	4 (19)	36 (45)	
CT pattern	Haziness	1 (4.8)	2 (2.5)	<0.001
	Nodular	7 (33.3)	31 (38.8)	
	Omental cake	0 (0)	18 (22.5)	
	Cystic	0 (0)	2 (2.5)	
	Mixed	2 (9.5)	20 (25)	
	Smooth	11 (52.4)	2 (2.5)	
	Mass	0 (0)	5 (6.3)	
Location of involvement	RUQ	5 (23.8)	9 (11.3)	0.069
	LUQ	0 (0)	15 (18.8)	
	Right paracolic	5 (23.8)	7 (8.8)	
	Right paracolic	1 (4.8)	2 (2.5)	
	Pelvis	2 (9.5)	5 (6.3)	
	Diffuse	8 (38.1)	42 (52.5)	
Lymphadenopathy		6 (28.6)	35 (48.3)	0.207
Ascites	Clear	3 (14.3)	12 (15)	<0.001
	Dense	9 (42.9)	62 (77.5)	
	No ascites	9 (42.9)	6 (7.5)	
Obesity		7 (33.3)	26 (32.5)	0.942

*Note*: The data are presented in frequency and %. The significance level was set as <0.05.

Abbreviations: CT, computed tomography; LUQ, left upper quadrant; RUQ, right upper quadrant.

The correlation between CT scans and ultrasound examinations in terms of diagnostic accuracy exhibited notable disparities for some medical conditions. For the colorectal adenocarcinoma, adenocarcinoma of the ovaries/uterus, and peritonitis, the statistical test results indicated a significant difference between the matched and non‐matched cases. (*p* < 0.001). Most of the cases which CT scan findings were not visible in ultrasound were patients with peritonitis. The relationships of other diseases exhibited considerable variability, and several diagnoses were characterized by insufficient cases to achieve statistical significance (Table 4).

**Table 4:** The association between CT scan and ultrasound matched findings with the final diagnosis.

Diagnosis	Nonmatched cases	Matched cases	*p* Value
Colorectal adenocarcinoma	4 (19)	16 (20)	<0.001
Adenocarcinoma of the ovaries/uterus	3 (14.3)	16 (20)	<0.001
Breast cancer	0 (0)	5 (6.3)	
Sarcoma of the uterus	0 (0)	5 (6.3)	
GTN	0 (0)	1 (1.3)	
Ovarian fibrothecoma	0 (0)	1 (1.3)	
GIST	0 (0)	2 (2.5)	
Gastric adenocarcinoma	0 (0)	20 (25)	
Peritonitis	10 (47.6)	1 (1.3)	<0.001
Esophageal adenocarcinoma	0 (0)	1 (1)	
Pancreatic adenocarcinoma	1 (4.8)	2 (2.5)	
Testicular rhabdomyosarcoma	0 (0)	1 (1.3)	
Sarcomatoid mesothelioma	0 (0)	1 (1.3)	
Small cell lung cancer	0 (0)	1 (1.3)	
Adenocarcinoma of the appendix/ilium	0 (0)	2 (2.5)	
Cholangiocarcinoma	1 (4.8)	2 (2.5)	
HCC	0 (0)	1 (1.3)	
Lymphoma	1 (4.8)	1 (1.3)	
Adenocarcinoma/TCC of the urinary tract	0 (0)	3 (3.8)	
TB	1 (4.8)	1 (1.3)	
SCC teratoma	0 (0)	1 (1.3)	

*Note*: The data are presented in frequency and %. The significance level was set as <0.05.

Abbreviations: CT, computed tomography; GIST, gastrointestinal stromal tumor; GTN, gestational trophoblastic neoplasia; HCC, hepatocellular carcinoma; SCC, squamous cell carcinoma; TB, tuberculosis; TCC, transitional cell carcinoma.

The correlation between CT scan patterns and diagnostic outcomes exhibited diverse patterns across several cancer types. The detailed results are shown in Table 5.

**Table 5:** The association between CT scan patterns and the diagnoses.

	Haziness	Mass, cyst, nodule	Omental cake	Mixed	Smooth
Colorectal adenocarcinoma	0 (0)	16 (80)	3 (15)	1 (5)	0 (0)
Gastric adenocarcinoma	2 (10)	5 (25)	6 (30)	7 (35)	0 (0)
GIST	0 (0)	2 (100)	0 (0)	0 (0)	0 (0)
Peritonitis	0 (0)	0 (0)	0 (0)	0 (0)	11 (100)
Esophageal adenocarcinoma	0 (0)	1 (100)	0 (0)	0 (0)	0 (0)
Pancreatic adenocarcinoma	0 (0)	1 (33.3)	1 (33.3)	1 (33.3)	0 (0)
Adenocarcinoma of the ovaries/uterus	1 (5.3)	8 (42.1)	3 (15.8)	6 (31.6)	1 (5.3)
Testicular rhabdomyosarcoma	0 (0)	0 (0)	1 (100)	0 (0)	0 ((0)
Breast cancer	0 (0)	1 (100)	0 (0)	0 (0)	0 (0)
Sarcoma of the uterus	0 (0)	3 (60)	0 (0)	2 (40)	0 (0)
Sarcomatoid mesothelioma	0 (0)	0 (0)	0 (0)	1 (100)	0 (0)
Small cell lung cancer	0 (0)	1 (100)	0 (0)	0 (0)	0 (0)
Adenocarcinoma of the appendix/ilium	0 (0)	1 (50)	1 (50)	0 (0)	0 (0)
Cholangiocarcinoma	0 (0)	1 (33.3)	1 (33.3)	1 (33.3)	0 (0)
GTN	0 (0)	0 (0)	0 (0)	1 (100)	0 (0)
HCC	0 (0)	1 (100)	0 (0)	0 (0)	0 (0)
Lymphoma	0 (0)	1 (50)	0 (0)	1 (50)	0 (0)
Adenocarcinoma/TCC of the urinary tract	0 (0)	2 (66.7)	1 (33.3)	0 (0)	0 (0)
TB	0 (0)	0 (0)	0 (0)	1 (50)	1 (50)
SCC teratoma	0 (0)	1 (100)	0 (0)	0 (0)	0 (0)

*Note*: The data are presented in frequency and %.

Abbreviations: CT, computed tomography; GIST, gastrointestinal stromal tumor; GTN, gestational trophoblastic neoplasia; HCC, hepatocellular carcinoma; SCC, squamous cell carcinoma; TB, tuberculosis; TCC, transitional cell carcinoma.

## 4 DISCUSSION

In our study, the most common peritoneal involvement patterns in CT scans included nodular, mixed, and omental cake, respectively. In 79.2% of patients, CT scan findings were consistent with ultrasound, but in 20.8%, CT scan findings were not visible in ultrasound. The most common diagnoses of the patients were colorectal adenocarcinoma and gastric adenocarcinoma, followed by ovarian and uterine adenocarcinoma.

The CT scan is a commonly employed imaging modality for various medical concerns. One illustrative instance involves the utilization of CT scanning for the evaluation of abdominal pain, a practice that has gained prominence, particularly in the availability of 24‐h CT scanners in emergency departments.^8^ CT offers a prompt diagnostic evaluation with exceptional visualization of the abdomen, together with a high degree of sensitivity.^9^, ^10^ Consequently, there has been a substantial rise of more than 330% in the utilization of CT in emergency medical settings throughout the period spanning from 1996 to 2007.^11^ Presently, it is expected that a proportion exceeding 16% of individuals who seek medical attention at the emergency department ED have a CT scan.^12^ The advantages of emergency CT scanning have been extensively documented in the literature. It has been shown that the incidence of harmful surgical interventions for patients suffering from acute appendicitis has decreased significantly, from 20% to below 4%.^13^ Furthermore, the reduction in missed diagnoses has led to a significant decrease in the length of hospital stays, as evidenced by earlier diagnoses.^14^, ^15^ In addition to the advantages above, the use of emergency CT has expanded due to a desire to mitigate risks and address concerns over the potential oversight of pathological conditions.^16^

This study aimed to examine the aetiology and distribution of omental and peritoneal involvement using CT scans and compare these findings with ultrasound results. The study population has a similar characteristic, as evidenced by the high incidence of clear ascites in 70.3% of cases and thick ascites in 14.9%. The presence of ascites, particularly when a bloody appearance characterizes it, has been seen to be potentially indicative of cancer,^17^ hence highlighting its significance as a diagnostic marker. The findings derived from our study indicate that the predominant manifestation observed in the CT scans conducted was the nodular pattern, which was identified in around 38% of the patient population. The mix pattern emerged as the second most prevalent pattern, accounting for 22% of the examined cases. Subsequently, the omental cake and smooth patterns were identified in 18% and 13% of the cases, respectively. The study done by Lee et al. yielded varying outcomes. The researchers demonstrated that the prevailing pattern of involvement seen was omental cake and nodular, as indicated by previous studies.^18^ It is important to acknowledge that the study conducted by the researchers focused only on individuals diagnosed with TB, which may account for the observed outcome variations. The prevalent pattern of participation in terms of location was the diffuse pattern found in around 50% of the patients. Subsequently, there was a higher prevalence of involvement in the left upper quadrant, right upper quadrant, and right paracolic regions compared to other areas. Conversely, there were very few reported cases of involvement in the pelvic and left paracolic regions.

In 40.6% of cases, lymphadenopathy was found to be positive, while metastasis was observed in 32.7%. Peritoneal lymphomatosis refers to the dissemination of lymphoma within the peritoneal cavity. The peritoneal lining does not possess lymphoid tissue. The issue surrounding the mechanism by which lymphoma infiltrates the peritoneum is a subject of debate among the academic community. According to a contemporary hypothesis, it is postulated that lymphoma dissemination occurs via the gut epithelium through ligaments and peritoneal folds. Other solitary or supplementary observations consist of the augmentation and linear or nodular thickening of the peritoneal lining, the presence of nodules in the omentum, and the occurrence of masses in the small bowel or colon.^19^

In contrast, the incidence of GIST results was seen in a mere 2% of the patients. The clinical manifestation of GIST sarcomatosis is characterized by many highly vascularized, diverse, and necrotic lesions, with a few occurrences of ascites.^20^ Ascites have been observed as a potential side effect in patients undergoing treatment with tyrosine kinase inhibitors. The complications associated with GIST sarcomatosis include gastrointestinal hemorrhage, hemoperitoneum, and fistulization.^21^

Moreover, TB was detected in 2% of the observed cases. Tuberculous peritonitis is uncommon. The peritoneum is affected by extrapulmonary TB in a mere 2% of cases.^22^ Several ideas exist that elucidate the mechanisms by which Mycobacterium TB disseminates to the peritoneum. The prevailing idea suggests that peritoneal TB is a result of the reactivation of latent foci that were previously created. Alternative ideas propose the occurrence of hematogenous dissemination from primary sites located either in close proximity or at a distance, as well as the release of cancer cells from affected mesenteric lymph nodes.^23^ There are three distinct classifications of tuberculous peritonitis, which are determined by the examination of CT scans and the overall visual appearance. It is worth noting that these classifications may or may not exhibit overlapping characteristics.^24^ The wet variety, which accounts for approximately 90% of cases, is distinguished by the presence of a substantial volume of ascitic fluid. Ascites can manifest as either free or loculated. The fluid exhibits considerable attenuation due to elevated protein and biological components levels. In contrast to ascites caused by carcinomatosis, the attenuation often exhibits a lower value. The occurrence of chylous ascites has been documented in correlation with tuberculous peritonitis. The second category is referred to as the fibrotic‐fixed form, accounting for around 7% of cases.^22^ This particular variety is distinguished by the presence of immobile intestinal loops, masses in the omentum, and a limited amount of ascitic fluid. The final and least prevalent category is the dry kind, accounting for only 3% of cases.^22^ This particular category encompasses the presence of thick peritoneal adhesions, fibrous peritoneal reaction, and caseous nodules. The characteristic that remains constant throughout all variations is the uniform and substantial thickening of the peritoneal lining. This characteristic differs from the nodular thickening observed in cases of peritoneal carcinomatosis.^22^, ^25^ The presence of omental caking is often not observed in cases with tuberculous peritonitis. The ultrasound examination reveals the presence of peritoneal thickening measuring between 2 and 6 mm and the observation of small nodules measuring less than 5 mm.^26^

The observed pattern of engagement in the CT scans demonstrates a wide array of manifestations. The most prevalent pattern observed in this study is mass, nodule, or cyst, accounting for 44.5% of cases. This is followed by mixed patterns, which comprise 21.7% of cases. The omental cake is the next most frequent pattern, representing 17.8% of cases. Smooth involvement is observed in 12.8% of cases, while haziness is the least common pattern, accounting for just 2.9% of cases. Identifying various patterns can offer valuable insights for distinguishing between different cancer kinds and stages, aiding in the process of differential diagnosis.

In colorectal adenocarcinoma and adenocarcinoma of the ovaries/uterus, ultrasound was able to show the findings of peritoneal involvement almost as well as CT scan. Ultrasound due to its characteristics, including the high‐resolution images, the absence of radiation, and the possibility of performing it at the bedside can be used as a first diagnostic imaging step for mentioned diseases. In contrast, most of the cases which CT scan findings were not visible in ultrasound were patients with peritonitis and ultrasound may not show the findings of peritoneal involvement in peritonitis well, thus it is not a suitable modality to investigate this pathology. However, due to small sample size in our research, further studies are required to determine more aspects of ultrasound efficacy in these conditions.

The correlation between CT scan results and patient attributes or ultimate diagnosis offers significant clinical information. The observed correlation between clear ascites and thick ascites and final diagnosis implies that the characteristics of ascites may serve as a dependable signal for particular forms of cancer. An illustration of this may be seen in the frequent correlation between thick ascites and progressive peritoneal carcinomatosis in instances of peritoneal surface cancers. Additionally, the investigation evaluated the correlation between gender and CT scan results. The discovery that females had a greater percentage of matched instances is a noteworthy result that merits more examination. The potential factors that may contribute to this phenomenon include variations in the incidence rates of certain cancer types between males and females, as well as disparities in the manner in which the illness manifests.

The findings of this investigation carry significant clinical consequences. Radiologists and oncologists can receive guidance from AI systems in interpreting CT scan results for patients who exhibit abdominal complaints. Identifying correlations between particular CT scan patterns and distinct cancer types might be beneficial in facilitating timely detection and formulating effective strategies for therapy. Additionally, the findings underscore the need to take into account the specific attributes of ascites and the site of their occurrence throughout the diagnostic procedure. A comprehensive assessment of these criteria has the potential to improve the precision of cancer diagnosis and facilitate the customization of treatment approaches for individual patients.

## 5 CONCLUSION

In summary, the aforementioned data completely examine CT scan observations, ultimate diagnoses, and their correlations within the studied population. The authors highlight the importance of accurate interpretation of CT scans in cancer diagnosis and stress the need for more research to gain a deeper understanding of the clinical implications of the specific patterns and relationships observed in this study. This knowledge can potentially enhance the efficacy and customization of cancer care for individuals.

## AUTHOR CONTRIBUTIONS

**Farzaneh Khoroushi**: Conceptualization; writing—original draft; data curation; project administration; supervision. **Lida Jarahi**: Formal analysis; methodology; investigation; validation. **Ehsan Hassannejad**: Writing—original draft; data curation; writing—review and editing; methodology. **Nafise Kazemirad**: Conceptualization; writing—original draft; writing—review and editing; data curation; formal analysis; methodology. All authors have read and approved the final version of the manuscript. Nafise Kazemirad had full access to all of the data in this study and takes complete responsibility for the integrity of the data and the accuracy of the data analysis.

## CONFLICT OF INTEREST STAETMENT

The authors declare no conflict of interest.

## ETHICS STATEMENT

The method was approved in compliance with scientific and ethical standards. All methods were performed in line with the relevant guidelines and regulations. The Medical Ethics Committee of Mashhad University of Medical Science approved this study.

## TRANSPARENCY STATEMENT

The Farzaneh Khoroushi affirms that this manuscript is an honest, accurate, and transparent account of the study being reported; that no important aspects of the study have been omitted; and that any discrepancies from the study as planned (and, if relevant, registered) have been explained.

## Data Availability

The data sets created during the current study are not publicly accessible due to the possibility of compromising the privacy of individuals.
